# Sinus computed tomography findings in patients with COVID-19

**DOI:** 10.31744/einstein_journal/2021AO6255

**Published:** 2021-03-09

**Authors:** Daniel Vaccaro Sumi, Rafael Maffei Loureiro, Simon Michael Collin, Patrícia Duarte Deps, Lorena Lima Bezerra, Regina Lúcia Elia Gomes, Mauro Miguel Daniel

**Affiliations:** 1 Hospital Israelita Albert Einstein São PauloSP Brazil Hospital Israelita Albert Einstein, São Paulo, SP, Brazil.; 2 Public Health England London United Kingdom Public Health England, London, United Kingdom.; 3 Universidade Federal do Espirito Santo VitóriaES Brazil Universidade Federal do Espirito Santo, Vitória, ES, Brazil.

**Keywords:** Tomography, X-Ray computed, Coronavirus infections, COVID-19, Betacoronavirus, SARS-CoV-2, Paranasal sinuses, Rhinitis

## Abstract

**Objective:**

To analyze computed tomography scans of paranasal sinuses of a series of patients with coronavirus disease 2019, and correlate the findings with the disease.

**Methods:**

Computed tomography scans of 95 adult patients who underwent a polymerase chain reaction test for severe acute respiratory syndrome coronavirus 2 were analyzed. Clinical data were obtained from patients’ records and telephone calls. Paranasal sinus opacification was graded and compared according to severe acute respiratory syndrome coronavirus 2 positivity.

**Results:**

Of the patients 28 (29.5%) tested positive for severe acute respiratory syndrome coronavirus 2 (median age 52 [range 26-95] years) and 67 were negative (median age 50 [range 18-95] years). Mucosal thickening was present in 97.4% of maxillary sinuses, 80% of anterior ethmoid air cells, 75.3% of posterior ethmoid air cells, 74.7% of frontal sinuses, and 66.3% of sphenoid sinuses. Minimal or mild mucosal thickening (score 1)and normally aerated sinuses (score 0) corresponded to 71.4% and 21.3% of all paranasal sinuses, respectively. The mean score of each paranasal sinus among severe acute respiratory syndrome coronavirus 2 positive and negative patients was 0.85±0.27 and 0.87±0.38, respectively (p=0.74). Median paranasal sinus opacification score among severe acute respiratory syndrome coronavirus 2 positive patients was 9 (interquartile range 8-10) compared to 9 (interquartile range 5-10) in negative patients (p=0.89). There was no difference in mean score adjusted for age and sex. Nasal congestion was more frequent in severe acute respiratory syndrome coronavirus 2 positive than negative patients (p=0.05).

**Conclusion:**

Severe acute respiratory syndrome coronavirus 2 infection was associated with patient recall of nasal congestion, but showed no correlation with opacification of paranasal sinuses.

## INTRODUCTION

Coronavirus disease 2019 (COVID-19) caused by severe acute respiratory syndrome coronavirus 2 (SARS-CoV-2) spread rapidly worldwide from its origin in China. Upper airway symptoms, such as nasal congestion and rhinorrhea, have been reported to be relatively rare when compared with pulmonary symptoms,^(^^1^^–^^3^^)^ and few studies have addressed the effects of SARS-CoV-2 infection on the paranasal sinuses using computed tomography (CT) scans.^(^^4^^–^^6^^)^

## OBJECTIVE

To analyze computed tomography scans of paranasal sinuses of a series of patients with coronavirus disease 2019, and correlate the findings with the disease.

## METHODS

### Patients

All patients undergoing paranasal sinus CT scans in our organization and who were tested for SARS-CoV-2 over an 11-week period, from March 14 to May 30, 2020, were eligible for inclusion. Emergency room, inpatients, and outpatients were included. Patient clinical and image data were reviewed retrospectively. All SARS-CoV-2 positive and a random sample of 30 SARS-CoV-2 negative patients were called by telephone. Those who responded were asked to recall whether they had anosmia, hyposmia and nasal congestion at the time of the CT scans. Severe acute respiratory syndrome coronavirus 2 positivity was determined from nasopharyngeal swabs collected for each patient and tested for SARS-CoV-2 using a real-time reverse transcription polymerase chain reaction (RT-PCR) assay.

The exclusion criteria were patients under 18 years of age; recent sinonasal surgery; sinonasal tumor; acute facial trauma.

### Imaging analysis

All patients underwent paranasal sinus CT scans (16- to 640-slice CT scanners: Aquilion Prime™, Aquilion One™, Aquilion One Vision™, Toshiba Medical Systems Corporation, Ltda, Japan; PET-CT Discovery 600, GE Medical Systems, USA; and SOMATOM^®^ Definition, Siemens, Germany) with the following parameters: 35mA, 120kV, 0.5 to 0.6mm slice thickness. All CT images were independently analyzed by two head and neck radiologists, with 5 and 12 years of experience, who were blinded to RT-PCR results. Disagreements were resolved by consensus.

Maxillary, anterior ethmoid, posterior ethmoid, sphenoid, and frontal paranasal sinuses were assessed for opacification. Sinuses with mucosal thickening <1mm were considered normal. Each paranasal sinus was graded according to a modified Lund-Mackay score as no opacification (score 0); opacification 1% to 50% (score 1); opacification 51% to 99% (score 2), and complete opacification (score 3). The presence ofair-fluid level was also evaluated.

### Statistical analysis

Frequencies and median scores in SARS-CoV-2 positive and negative patients were compared using *χ*^2^ or Fisher's exact, Kruskal-Wallis, and Student's *t* tests (alpha <0.05), while differences in total opacification score and olfactory obstruction, adjusted for age and sex, were quantified by logistic and linear regression, respectively.

This study was approved by the Ethics Committee of *Hospital Israelita Albert Einstein* (HIAE), CAAE: 31014720.9.0000.0071, number 4.002.511. The need for written patient consent was waived.

## RESULTS

A total of 95 patients were included, of whom 28 (29.5%) were SARS-CoV-2 RT-PCR-positive and 67 (70.5%) were negative. The median age was 50 (range 18 to 95) years. There were 53 men (55.8%). The two groups did not differ by age or sex (Table 1).

**Table 1:** Patient characteristics and paranasal sinus computed tomography findings by severe acute respiratory syndrome coronavirus 2 polymerase chain reaction test status

Characteristics	SARS-CoV-2 negative (n=67)	SARS-CoV-2 positive (n=28)	p value*
Age, years	50 (37-62)	52 (44-63)	0.34
Male sex (%)	38 (56.7)	15 (53.6)	0.78
Paranasal sinus opacification score	9 (5-10)	9 (8-10)	0.89
Air-fluid level (%)	14 (20.9)	6 (21.4)	0.95
Nasal obstruction† (%)	1/23 (4.3)	7/17 (41)	0.05

* median (interquartile range - IQR) and Kruskal-Wallis test for age and paranasal sinus opacification score (range 0-30); frequency (%) and *χ*^2^ test for sex, air-fluid level frequency (%) and Fisher's exact test for nasal obstruction;

† as reported by patients who were called by telephone.

SARS-CoV-2: severe acute respiratory syndrome coronavirus 2.

Among the 17/28 SARS-CoV-2 positive and 23/30 SARS-CoV-2 negative patients who responded to telephone follow-up, anosmia/hyposmia was recalled by 7/17 (41%) positive and 0/23 negative patients (*p* = 0.001). Nasal congestion was recalled by 7/17 (41%) SARS-CoV-2 positive and by 1/23 (4.3%) SARS-Cov-2 negative patients (p=0.05).

**Figure 1:**
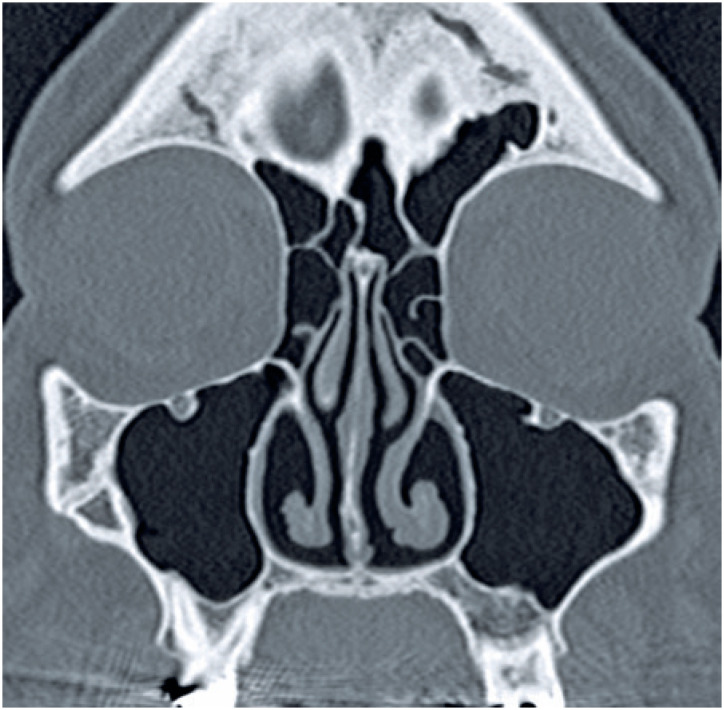
Coronal non-enhanced computed tomography scan of a severe acute respiratory syndrome coronavirus 2 positive patient shows normal aeration of the paranasal sinuses (score 0)

**Figure 2:**
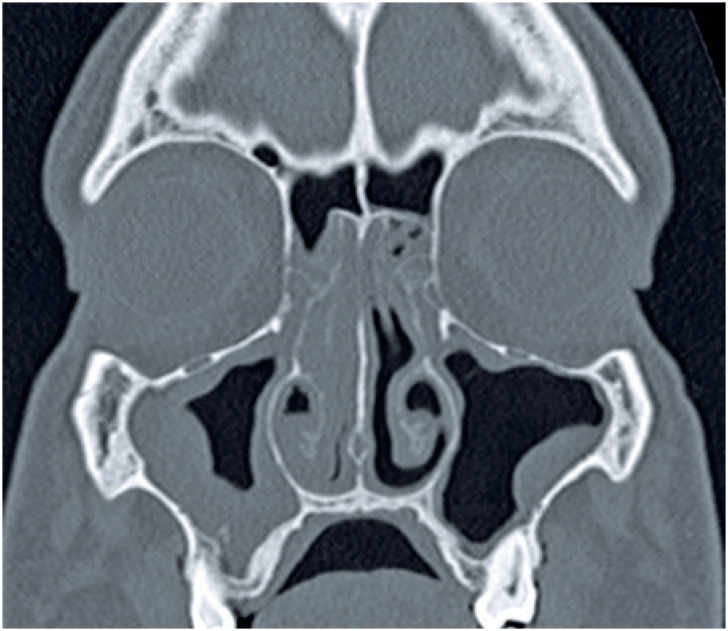
Coronal non-enhanced computed tomography scan of a severe acute respiratory syndrome coronavirus 2 negative patient shows mild mucosal thickening of the left maxillary sinus (score 1) and partial opacification (51% to 99%) of the right maxillary sinus (score 2)

Mucosal thickening was present in 97.4% of maxillary sinuses, 80% of anterior ethmoid air cells, 75.3% of posterior ethmoid air cells, 74.7% of frontal sinuses, and 66.3% of sphenoid sinuses. Minimal or mild mucosal thickening (score 1) and normally aerated sinuses (score 0); corresponded to 71.4% and 21.3% of all paranasal sinuses, respectively. Marked mucosal thickening (score 2) corresponded to 6.9% and complete opacification (score 3), 0.4% (Figures 1 and 2). No significant difference was observed among the paranasal sinuses (Table 2).

**Table 2:** Modified Lund-Mackay score of paranasal sinuses computed tomography scans in severe acute respiratory syndrome coronavirus 2 positive patients

Paranasal sinus	Right				Left			
	0	1	2	3	0	1	2	3
Maxillary	1/3	26/80	1/12	0	0/2	6/82	4/11	0
Ant ethmoid	5/21	23/67	0/7	0	5/17	22/66	1/11	0/1
Post ethmoid	9/26	19/62	0/6	0/1	6/21	22/71	0/3	0
Frontal	5/26	23/66	0/3	0	5/22	22/68	1/4	0/1
Sphenoid	10/33	18/58	0/3	0/1	6/31	20/58	2/6	0
Total (%)	30/109 (27.5)	109/333 (32.7)	1/31 (3.2)	0/2 (0)	22/93 (23.7)	92/345 (26.7)	8/35 (22.9)	0/2 (0)
Air-fluid level	Present (%)		Absent (%)					
	6/20 (30)		22/75 (29.3)					

The mean score of each paranasal sinus among SARS-CoV-2 positive and negative patients was 0.85±0.27 and 0.87±0.38, respectively (p=0.74).

The median paranasal sinus opacification score for each SARS-CoV-2 positive patient was 9 (interquartile range – IQR: 8-10) compared to 9 (IQR: 5-10) in negative patients (p=0.89). There was no difference in mean score adjusted for age and sex. Scores of 13 and higher were more frequent in 9/67 (13.4%) SARS-CoV-2 negative and in only 1/28 (3.6%) positive patients.

The median paranasal sinus score was ten, both among patients who reported anosmia/hyposmia (IQR: 8-11) and among those who did not report this symptom (IQR: 7-11; p=0.99) (Table 3).

**Table 3:** Median total opacification score in patients with and without recall of anosmia/hyposmia

Anosmia	p25	p50	p75	Total
Present	8	10	11	7
Absent	7	10	11	33

Median (interquartile range) and Kruskal-Wallis test.

Air-fluid level was present in 6/28 (21.4%) positive patients compared with 14/67 (20.9%) negative patients (p=0.95).

Air-fluid level was present in 6/28 (21.4%) positive patients compared with 14/67 (20.9%) negative patients (p=0.95).

## DISCUSSION

Sinonasal symptoms in SARS-CoV-2 positive patients are generally considered to be rare when compared to lower respiratory tract symptoms, although variations in prevalence (4% to 35% in the case of rhinorrhea) have been reported.^(^^1^^–^^3^^)^

Nasal congestion was reported by 41% of SARS-CoV-2 positive patients, which correlates with the findings of Lechien et al.,^(^^4^^)^ (36.9%) and Naeini et al.,^(^^5^^)^ (32.7%) notwithstanding the differences of methodology.

Few articles have addressed the effect of SARS-CoV-2 infection on the paranasal sinuses. Chung et al.,^(^^6^^)^ reported no signs of sinusitis on CT scans of six SARS-CoV-2 positive patients who had olfactory dysfunction. A recent article included 49 COVID-19 positive patients with anosmia, used the original Lund-Mackay scoring system, and showed partial opacification in less than 10% of maxillary, frontal and sphenoid sinuses, and the ethmoid air cells were normally aerated in all patients.^(^^5^^)^ In a cohort of 16 COVID-19 positive patients with olfactory dysfunction, Lechien et al.,^(^^4^^)^ also found no significant mucosal thickening of paranasal sinuses, with a Lund-Mackay mean score of 0.8.

Our study included 95 COVID-19 positive and negative patients. We decided to use a modified Lund-Mackay scoring system to better distinguish mild mucosal thickening from almost completely opacified sinuses. Despite the use of a modified scoring system, our findings corroborated the findings of these previous studies. We found similar scores for both SARS-CoV-2 positive and negative patients, with scores zero and one being the most common, with a median score of nine for both SARS-CoV-2 positive and negative patients (out of a maximum score of 30; p=0.89). Indeed, scores of 13 or higher were more frequent in SARS-CoV-2 negative patients. Our findings show that SARS-CoV-2 infection is not associated with mucosal changes in the paranasal sinuses.

There is scarce evidence in the literature regarding the effects of common viral respiratory infections in the paranasal sinuses. Gwaltney et al.,^(^^7^^)^ found opacification varying from 87% in the maxillary sinuses to 39% in the sphenoid sinuses among patients with common cold. Alho,^(^^8^^)^ using the Lund-Mckay scoring system, found CT mean scores from seven to ten among patients with fresh viral cold. Our results are similar to those obtained in these articles, both in SARS-CoV-2 positive and negative patients.

This was an observational study, therefore prone to selection bias if SARS-CoV-2 positive patients were more likely to undergo a CT scan for clinical reasons related to our outcomes. It also had a relatively small sample size which was determined by an 11-week period of recruitment rather than on any *a priori* knowledge of possible associations. Nasal congestion was assessed by asking participants to recall whether they had this symptom at the time of their CT scan, a method which is susceptible to recall bias. Clinical data such as the average duration of symptoms and the final diagnoses of patients who tested negative for SARS-CoV-2 infection were not available in most cases. Also, we did not extract follow-up information on the clinical course and eventual outcome of SARS-CoV-2 infection.

## CONCLUSION

SARS-CoV-2 infection was associated with patient recall of nasal congestion, but showed no correlation with opacification of paranasal sinuses.
