# Supplementary material for: Sinus computed tomography findings in patients with COVID-19
Source: Einstein (Sao Paulo). 2021 Mar 9;19:eAO6255. doi: 10.31744/einstein_journal/2021AO6255 (PMC7942839; doi:10.31744/einstein_journal/2021AO6255)
Supplement: Translation

# Translation: Achados de imagem em tomografias computadorizadas de seios paranasais em pacientes com COVID-19

**Authors:** Daniel Vaccaro Sumi, Rafael Maffei Loureiro, Simon Michael Collin, Patrícia Duarte Deps, Lorena Lima Bezerra, Regina Lúcia Elia Gomes, Mauro Miguel Daniel

**Affiliations:** 1 São PauloSP Brasil Hospital Israelita Albert Einstein, São Paulo, SP, Brasil.; 2 London United Kingdom Public Health England, London, United Kingdom.; 3 VitóriaES Brasil Universidade Federal do Espírito Santo, Vitória, ES, Brasil.

**Keywords:** Tomografia computadorizada por raios X, Infecções por coronavírus, COVID-19, Betacoronavírus, SARS-CoV-2, Seios paranasais, Rinite

## Abstract

**Objetivo:**

Analisar imagens de tomografia computadorizada de seios paranasais de pacientes com a doença por coronavírus 2019, e correlacionar os achados com a doença.

**Métodos:**

Foram analisadas imagens de tomografia computadorizada de 95 pacientes submetidos a teste de reação em cadeia da polimerase para coronavírus 2 da síndrome respiratória aguda grave. Os dados clínicos foram obtidos por meio dos prontuários dos pacientes e de ligações telefônicas. A opacificação dos seios paranasais foi graduada e comparada entre pacientes positivos e negativos para coronavírus 2 da síndrome respiratória aguda grave.

**Resultados:**

Vinte e oito (29,5%) dos pacientes tiveram resultado positivo para coronavírus 2 da síndrome respiratória aguda grave (idade mediana de 52 [26-95] anos) e 67, resultado negativo (idade mediana de 50 [18-95] anos). O espessamento mucoso estava presente em 97,4% dos seios maxilares, 80% das células etmoidais anteriores, 75,3% das células etmoidais posteriores, 74,7% dos seios frontais e em 66,3% dos seios esfenoidais. Mínimo ou discreto espessamento mucoso (pontuação 1) e seios com aeração normal (pontuação 0) corresponderam a 71,4% e 21,3% de todos os seios paranasais, respectivamente. A nota média de cada seio paranasal entre pacientes positivos e negativos para coronavírus 2 da síndrome respiratória aguda grave foi de 0,85±0,27 e 0,87±0,38, respectivamente (p=0,74). A nota mediana de opacificação dos seios paranasais entre pacientes positivos para coronavírus 2 da síndrome respiratória aguda grave foi de 9 (intervalo interquartil de 8 a 10), comparada a 9 (intervalo interquartil de 5 a 10) em pacientes negativos (p=0,89). Não houve diferença na nota média ajustada para idade e sexo. A congestão nasal foi mais frequente em pacientes positivos para coronavírus 2 da síndrome respiratória aguda grave que naqueles com resultados negativos (p=0,05).

**Conclusão:**

A infecção pelo coronavírus 2 da síndrome respiratória aguda grave apresentou associação com congestão nasal, mas não mostrou correlação com espessamento mucoso dos seios paranasais.

## INTRODUÇÃO

A doença pelo coronavírus 2019 (COVID-19) causada pelo coronavírus da síndrome respiratória aguda grave 2 (SARS-CoV-2) se espalhou rapidamente pelo mundo a partir de sua origem na China. Os sintomas das vias aéreas superiores, como congestão nasal e rinorreia, foram relatados como relativamente raros quando comparados aos sintomas pulmonares,^(^^1^^–^^3^^)^ e poucos estudos abordaram os efeitos da infecção por SARS-CoV-2 nos seios paranasais usando tomografia computadorizada (TC).^(^^4^^–^^6^^)^

## OBJETIVO

Analisar imagens de tomografia computadorizada de seios paranasais de pacientes com a doença por coronavírus 2019, e correlacionar os achados com a doença.

## MÉTODOS

### Pacientes

Todos os pacientes submetidos à TC de seios paranasais em nossa instituição e testados para SARS-CoV-2 durante um período de 11 semanas, de 14 de março a 30 de maio de 2020, foram elegíveis para inclusão. Foram incluídos pacientes atendidos no pronto-socorro, internados e ambulatoriais. Os dados clínicos e de imagem dos pacientes foram revisados retrospectivamente. Todos os pacientes com resultado positivo para SARS-CoV-2 e uma amostra aleatória de 30 pacientes com resultado negativo foram contatados por telefone. Os pacientes que atenderam a ligação, solicitou-se relembrar se tiveram anosmia, hiposmia e congestão nasal no momento da tomografia. A positividade para SARS-CoV-2 foi determinada a partir de *swabs* nasofaríngeos coletados para cada paciente e testados usando um ensaio de transcrição reversa seguida de reação em cadeia da polimerase em tempo real (RT-PCR).

Foram excluídos pacientes menores de 18 anos, que passaram por cirurgia nasossinusal recentemente, com tumor nasossinusal e trauma facial agudo.

### Análise de imagem

Todos os pacientes foram submetidos a TC de seios paranasais (tomógrafos de 16 a 640 cortes: Aquilion Prime™, Aquilion One™, Aquilion One Vision™, Toshiba MedicalSystems Corporation, Ltda, Japão; PET-CT Discovery 600, GE Medical Systems, EUA; e SOMATOM^®^ Definition, Siemens, Alemanha) com os seguintes parâmetros: 35mA, 120kV e espessura de corte de 0,5 a 0,6mm. Todas as imagens de TC foram analisadas de forma independente por dois radiologistas de cabeça e pescoço, com 5 e 12 anos de experiência, que desconheciam os resultados da RT-PCR. As discordâncias foram resolvidas por consenso.

Os seios paranasais maxilares, etmoidais anteriores e posteriores, esfenoidais e frontais foram avaliados quanto à opacificação. Os seios paranasais com espessamento da mucosa <1mm foram considerados normais. Cada seio paranasal foi classificado de acordo com o sistema de pontuação de Lund-Mackay modificado como sem opacificação (pontuação 0); opacificação de 1% a 50% (pontuação 1); opacificação de 51% a 99% (pontuação 2) e opacificação completa (pontuação 3). A presença de nível hidroaéreo também foi avaliada.

### Análise estatística

As frequências e as pontuações medianas em pacientes positivos e negativos para SARS-CoV-2 foram comparadas por meio do teste do χ^2^ ou teste exato de Fisher, teste de Kruskal-Wallis e teste *t* de Student (alfa <0,05), ao passo que as diferenças na pontuação total de opacificação e obstrução olfatória, ajustadas para idade e sexo, foram quantificadas por regressão logística e linear, respectivamente.

Este estudo foi aprovado pelo Comitê de Ética do Hospital Israelita Albert Einstein (HIAE), com CAAE: 31014720.9.0000.0071, número 4.002.511. Houve dispensa de necessidade de consentimento por escrito dos pacientes.

## RESULTADOS

Foram incluídos 95 pacientes, dos quais 28 (29,5%) tiveram resultado positivo na RT-PCR para SARS-CoV-2 e 67 (70,5%) negativo. A idade mediana foi 50 (variação de 18 a 95) anos. Havia 53 homens (55,8%). Os dois grupos não diferiram quanto à idade ou ao sexo (Tabela 1).

**Tabela 1:** Características dos pacientes e achados na tomografia computadorizada dos seios paranasais por situação do teste de reação em cadeia da polimerase do coronavírus 2 da síndrome respiratória aguda grave

Características	SARS-CoV-2 negativo (n=67)	SARS-CoV-2 positivo (n=28)	Valor de p*
Idade, anos	50 (37-62)	52 (44-63)	0,34
Sexo masculino (%)	38 (56,7)	15 (53,6)	0,78
Pontuação de opacificação do seio paranasal	9 (5-10)	9 (8-10)	0,89
Nível hidroaéreo (%)	14 (20,9)	6 (21,4)	0,95
Obstrução nasal† (%)	1/23 (4,3)	7/17 (41)	0,05

* Mediana (intervalo interquartil-IIQ) e teste de Kruskal-Wallis para idade, e pontuação de opacificação dos seios paranasais (variação 0-30); frequência (%) e teste χ^2^ para sexo, frequência do nível hidroaéreo (%). Teste exato de Fisher para obstrução nasal;

† conforme relatado pelos pacientes contatados por telefone.

SARS-CoV-2: coronavírus da síndrome respiratória aguda grave 2.

Entre os 17 de 28 pacientes com resultado positivo para SARS-CoV-2 e em 23 de 30 negativos para SARS-CoV-2 contatados por telefone, anosmia/hi­posmia foi lembrada por 7 de 17 (41%) pacientes positivos e por nenhum dos 23 negativos (*p* = 0,001). Congestão nasal foi lembrada por 7 de 17 (41%) dos pacientes com resultado positivo para SARS-CoV-2 e por um dos 23 (4,3%) pacientes com resultado negativo SARS-Cov-2 (p=0,05).

O espessamento da mucosa estava presente em 97,4% dos seios maxilares, 80% das células etmoidais anteriores, 75,3% das células etmoidais posteriores, 74,7% dos seios frontais e 66,3% dos seios esfenoidais. O espessamento mínimo ou discreto da mucosa (pontuação 1) e seios paranasais com aeração normal (pontuação 0) corresponderam a 71,4% e 21,3% de todos os seios paranasais, respectivamente. O espessa­mento acentuado da mucosa (pontuação 2) ocorreu em 6,9% e a opacificação completa (pontuação 3), em 0,4% (Figuras 1 e 2). Não foi observada diferença significativa entre os seios paranasais (Tabela 2).

**Figura 1:** Tomografia computadorizada coronal sem contraste de um paciente positivo para coronavírus 2 da síndrome respiratória aguda grave mostra aeração normal dos seios paranasais (pontuação 0)

**Figura 2:** Tomografia computadorizada coronal sem contraste de um paciente negativo para coronavírus 2 da síndrome respiratória aguda grave mostra discreto espessamento da mucosa do seio maxilar esquerdo (pontuação 1) e opacificação parcial (51% a 99%) do seio maxilar direito (pontuação 2)

**Tabela 2:** Pontuação modificada de Lund-Mackay da tomografia computadorizada de seios paranasais em pacientes positivos para coronavírus 2 da síndrome respiratória aguda grave

Seios paranasais	Direito				Esquerdo			
	0	1	2	3	0	1	2	3
Maxilar	1/3	26/80	1/12	0	0/2	6/82	4/11	0
Etmoidal anterior	5/21	23/67	0/7	0	5/17	22/66	1/11	0/1
Etmoidal posterior	9/26	19/62	0/6	0/1	6/21	22/71	0/3	0
Frontal	5/26	23/66	0/3	0	5/22	22/68	1/4	0/1
Esfenoidal	10/33	18/58	0/3	0/1	6/31	20/58	2/6	0
Total (%)	30/109 (27,5)	109/333 (32,7)	1/31(3,2)	0/2 (0)	22/93 (23,7)	92/345 (26,7)	8/35 (22,9)	0/2 (0)
Nível hidroaéreo	Presente (%)		Ausente (%)					
	6/20 (30)		22/75 (29,3)					

A pontuação média de cada seio paranasal entre os pacientes positivos e negativos para SARS-CoV-2 foi de 0,85±0,27 e 0,87±0,38, respectivamente (p=0,74).

A pontuação mediana de opacificação dos seios paranasais para cada paciente com resultado positivo para SARS-CoV-2 foi 9 (intervalo interquartil – IIQ: 8-10) em comparação com 9 (IIQ: 5-10) em pacientes negativos (p=0,89). Não houve diferença na pontuação média ajustada para idade e sexo. Pontuações de 13 e superiores foram encontradas em 9 de 67 (13,4%) pacientes com resultado negativo para SARS-CoV-2 e em apenas um de 28 (3,6%) pacientes positivos.

A pontuação mediana dos seios paranasais foi dez, tanto entre os pacientes que relataram anosmia/hiposmia (IIQ: 8-11) quanto entre aqueles que não relataram esse sintoma (IIQ: 7-11; p=0,99) (Tabela 3).

**Tabela 3:** Pontuação total mediana de opacificação em pacientes com e sem lembrança de anosmia/hiposmia

Anosmia	p25	p50	p75	Total
Presente	8	10	11	7
Ausente	7	10	11	33

Mediana (intervalo interquartil) e teste de Kruskal-Wallis.

Nível hidroaéreo foi encontrado em 6 de 28 (21,4%) pacientes com resultados positivos em comparação com 14 de 67 (20,9%) pacientes com resultados negativos (p=0,95).

## DISCUSSÃO

Sintomas nasossinusais em pacientes positivos para SARS-CoV-2 são geralmente considerados raros quando comparados aos sintomas do trato respiratório inferior, embora tenham sido relatadas variações na prevalência (4% a 35%, no caso de rinorreia).^(^^1^^–^^3^^)^

A congestão nasal foi relatada por 41% dos pa­cientes com resultado positivo para SARS-CoV-2, em correlação com os achados de Lechien et al.,^(^^4^^)^ (36,9%), e Naeini et al.,^(^^5^^)^ (32,7%), apesar das diferenças de metodologia.

Poucos artigos abordaram o efeito da infecção por SARS-CoV-2 nos seios paranasais. Chung et al.,^(^^6^^)^ não relataram sinais de sinusite em exames de TC de seis pacientes positivos para SARS-CoV-2 que apresentavam disfunção olfatória. Artigo recente, que incluiu 49 pacientes positivos para COVID-19 com anosmia e que usou o sistema de pontuação Lund-Mackay original, mostrou opacificação parcial em menos de 10% dos seios maxilares, frontais e esfenoidais, e as células etmoi­dais estavam normalmente aeradas em todos os pacientes.^(^^5^^)^ Em uma coorte de 16 pacientes positivos para COVID-19 com disfunção olfatória, Lechien et al.^(^^4^^)^ também não encontraram espessamento mucoso significativo dos seios paranasais, com pontuação média de Lund-Mackay de 0,8.

Nosso estudo incluiu 95 pacientes positivos e negativos para COVID-19. Decidimos usar um sistema de pontuação de Lund-Mackay modificado, para diferenciar melhor o espessamento leve da mucosa e a opacificação quase completa dos seios paranasais. Apesar do uso de um sistema de pontuação modificado, nossos achados corroboram os desses estudos anteriores. Encontramos pontuações semelhantes para pacientes com resultado positivo e negativo para SARS-CoV-2, sendo as pontuações 0 e 1 as mais comuns e uma pontua­ção mediana de nove para pacientes positivos e negativos para SARS-CoV-2 (de uma pontuação máxima de 30; p=0,89). De fato, as pontuações maiores ou iguais a 13 foram mais frequentes em pacientes com resultado negativo para SARS-CoV-2. Nossos achados mostram que a infecção por SARS-CoV-2 não está asso­ciada a alterações na mucosa dos seios paranasais.

Existem poucas evidências na literatura sobre os efeitos das infecções respiratórias virais comuns nos seios paranasais. Gwaltney et al.,^(^^7^^)^ encontraram opacificação que variava de 87%, nos seios maxilares, a 39%, nos seios esfenoidais, em pacientes com resfriado comum. Alho,^(^^8^^)^ usando o sistema de pontuação deLund-Mackay, encontrou pontuações médias na TC de sete a dez em pacientes com resfriado viral recente. Nossos resultados são semelhantes aos obtidos nesses estudos, tanto em pacientes com resultado positivo quanto negativo para SARS-CoV-2.

Nosso estudo foi observacional, estando, portanto, sujeito a viés de seleção, caso os pacientes positivos para SARS-CoV-2 tenham ficado mais propensos a serem submetidos a uma TC por razões clínicas relacionadas aos nossos desfechos. Além disso, tinha um tamanho de amostra relativamente pequeno, determinado por um estudo com um período de recrutamento de 11 semanas e não por qualquer conhecimento *a priori* de possíveis associações. A congestão nasal foi avaliada por uma solicitação aos participantes de que relembrassem se eles tinham esse sintoma no momento da TC, um método que é suscetível a viés de memória. Os dados clínicos, como a duração média dos sintomas e os diagnósticos finais dos pacientes com resultado negativo para infecção por SARS-CoV-2, não estavam disponíveis na maioria dos casos. Além disso, não extraímos informações de acompanhamento sobre o curso clínico e o desfecho final da infecção por SARS-CoV-2.

## CONCLUSÃO

A infecção pelo SARS-CoV-2 foi associada com a lembrança do paciente de ter tido congestão nasal, mas não mostrou correlação com a opacificação dos seios paranasais.
